# Self-assembled monolayer of poly(*o*-phenylenediamine)/silver core–shell hybrid-based enzyme-free impedimetric glucose sensor for blood samples†

**DOI:** 10.1039/d4ra04766d

**Published:** 2024-08-27

**Authors:** Mukul Deo, Devleena Sahoo, Pradip Kar

**Affiliations:** a Department of Chemistry, Birla Institute of Technology Mesra Ranchi-835215 Jharkhand India pkar@bitmesra.ac.in pradipkgp@gmail.com

## Abstract

A core–shell hybrid of poly(*o*-phenylenediamine)/silver was prepared by a simple single-step process and self-assembled on a glassy carbon electrode to design an enzyme-free electrochemical glucose sensor. The working electrode was fabricated by self-assembling a dimethyl sulfoxide (DMSO) solution of the prepared hybrid on a glassy carbon electrode. The electrochemical properties of the fabricated electrode were analyzed by cyclic voltammetry (CV) and electrochemical impedance spectroscopy (EIS) in an aqueous potassium chloride electrolyte containing the [Fe(CN)_6_]^3−/4−^ redox couple. The electrochemical sensing responses towards varying concentrations of glucose were studied by CV and EIS in the same redox electrolyte medium. The poly(*o*-phenylenediamine)/silver core–shell hybrid-based sensor was found to show a reliable response in the EIS experiment over the CV experiment. By analyzing the EIS sensing responses, the experimental limit of detection was determined to be 10 μL of ∼80 mg per dL glucose solution in 25 mL of 1 (M) KCl electrolyte containing the 10^−3^ M [Fe(CN)_6_]^3−/4−^ redox couple with a sensitivity of 298 Ω mg^−1^ dL cm^−2^. Excellent selectivity towards glucose was confirmed over various bioactive interfering biomolecules like ascorbic acid, dopamine, uric acid, lactose, fructose and sucrose. The glucose content in the human blood sample was verified by the designed sensor with almost 100% recovery.

## Abstract 2

A core–shell hybrid of poly(*o*-phenylenediamine)/silver was prepared by a simple single-step process and self-assembled on a glassy carbon electrode to design an enzyme-free impedimetric glucose sensor.

## Graphical abstract

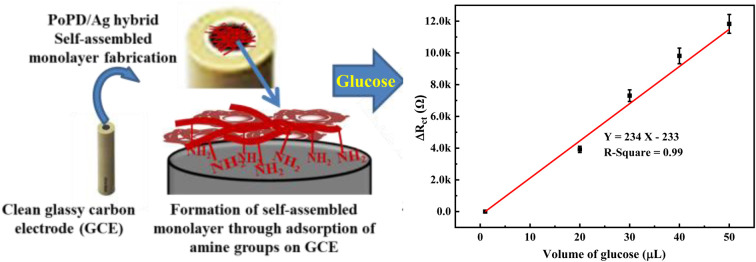

## Introduction

For the mammalian brain, glucose is a necessary metabolite to produce energy and around 20% of this energy is consumed by the brain. Therefore, an abnormal glucose concentration level indicates an anomaly in the normal metabolism, which leads to neurological diseases like diabetes and metabolic disorders. Diabetes is one of the most prevalent chronic diseases among the elderly, adults, young adults, and even children, rising at a speculative rate of more than 75% in the last 6 years. Since the last two decades, the mortality rate of the population due to diabetes has elevated by 3%, which is an affliction for the world. Besides diabetes being an autoimmune disorder, it also invites diseases like retinopathy, kidney and liver issues, non-healing wounds and cardiovascular diseases. The need of the hour is to introduce devices that are easily accessible and enable early diagnosis of diabetes, while also encouraging necessary changes into modern lifestyles. Consequently, monitoring glucose levels becomes extremely important to take proper prevention before the situation worsens. The sensitive and selective sensing of glucose is not only relevant for use in clinical diagnosis but also in the food industry, bio-processing and safety, drug discovery, environmental monitoring, fermentation monitoring, development of renewable, sustainable fuel cells, *etc.*

Many complicated, costly and less efficient strategies for the detection of glucose have been investigated. Here, electrochemical methods are the most effective because of their high sensitivity, convenience, speed, and financial viability. With time and the application of enzymatic glucose sensors in the real world, their usage became obscure as other bioactive compounds (uric acid, ascorbic acid) were found to interfere with the detection of glucose at a similar potential range. Moreover, the enzymatic electrochemical sensors generally suffer from unavoidable problems like the inefficiency of enzymes at the assay environmental conditions, *e.g.*, temperature and pH value, poor stability and inactivity of the enzyme, poor charge transfer to the electrode surface, and very poor long-term accuracy. In addition, the commercially-available glucose sensors are enzyme-based and do not fulfil the minimum standard criteria, *e.g.*, ISO 15197:2013.^1^ Several other electrochemical methods of blood glucose detection have been introduced by altering the material on the electrodes, altering the methods of fabrication, altering the immobilization techniques, *etc.* The research in this area has seen a more remarkable shift over the last four/five decades towards fourth-generation biosensors. In the fourth-generation electrochemical glucose sensor, the direct oxidation of glucose on the electrode is clearly superior to the enzymatic one in terms of the high sensitivity, good stability, reproducibility, and strong anti-interference properties. As per the literatures, the nanomaterials of various metals like Pt, Au, Cu, Ni, Pd, Ti, and Fe, metals oxides (TiO_2_, Mo_2_O_3_, NiO, RuO_2_, CuO, Co_3_O_4_, ZnO, ZrO_2_, *etc.*)^2,3^ and carbon can be utilized to fabricate non-enzymatic electrodes for the successful sensing of different biomolecules^4–6^ by cyclic voltammetry. Mostly, metal nanoparticles like Pt, Au, Ag, and Pd or their respective alloys or mixtures have been exploited as mediators for the successful sensing of glucose.^7–9^ An aqueous alkaline medium is generally required for the electrocatalytic oxidation of glucose on the hydroxide layer of these metal nanomaterials and thus restricts their lifetime. In this context, impedimetric glucose sensors are drawing recent interest due to their high precision, stability, affinity sensing, use in non-basic medium, *etc.*^10–16^ The utility and synergy of conjugated polymeric materials with metal nanoparticles or metal frameworks has been proven to be entirely operational for detecting specific moieties.^17,18^ This perspective shows that conjugated polymer-based metal nanocomposites have numerous advantages, *viz.*, excellent reproducibility, easy fabrication, better selectivity and, most importantly, increased sensitivity, making them a suitable choice for non-enzymatic biosensing materials.^15–20^ Moreover, the electrode fabrication methods like drop casting, spin coating, and dip coating were found to be responsible for such poor stability including inconsistent electrochemical performances and less reproducible sensing response. Likewise, the metal nanomaterials-based electrode fabricated by the same methods exhibited limited selectivity and sensitivity including their poor reproducibility, poor repeatability, high expensiveness, chloride ions accumulations, adsorption, and loss of intermediates. Here, the electrode modification by self-assembled monolayer formation should be given importance in order to have steady electrochemical characteristics and reproducible sensing performances. Owing to their high electronic conduction, excellent electrocatalytic behaviour, good chemical stability, biocompatibility, low toxicity, ease of synthesis from less costly stable salt, *etc.*, silver nanoparticles have attracted the wide interest of researchers among metal nanoparticles to develop the potential biosensors.^21,22^ In this context, polyaniline or its derivative-based composite with metal nanoparticles has been extensively used to develop biosensors including glucose sensors.^15,17,23,24^ This is because of the potential advantages of polyaniline or its derivatives as the electrode material like good electroactivity, extended π-conjugation, easily reversible redox switching, and reversible doping/de-doping behaviour to produce the electrochemical signal during glucose sensing. However, the conjugated polymer-based glucose sensors suffer from serious fundamental/technical issues like (i) insolubility in organic solvent, obstructing the fabrication of the working electrode, (ii) instability of the dopant in an aqueous medium, (iii) low electroactivity without doping, restricting the electron transfer, (iv) irreversible reduction of an oxidized conjugated ring in the aqueous basic medium, (v) relatively high detection limit with low selectivity, *etc.* There are immune scopes for the further improvement of glucose sensing performances using some active conjugated polymer-based materials. Here, the polyaniline derivative containing active functional groups such as amine or hydroxyl would have a better prospect as the electrochemical bio-sensor material due to easy redox switching, better electroactivity, superior interfacial interaction, and solution processability.^25,26^ Solution-processable poly(*o*-phenylenediamine) (PoPD) containing either the –NH_2_ or =NH groups have been introduced as promising electroactive materials with better electronic conduction.^27^ Moreover, easy and reliable self-assembly fabrication methods might be adopted using the free functional groups of the polymer. Recently, we have also reported the successful preparation of the processable poly(*o*-phenylenediamine)/silver (PoPD/Ag) core–shell hybrid by the single-step method.^28^ As revealed, the doping of the PoPD matrix by the strong electrostatic interfacial interaction of silver nanoparticles would be useful as an electronic material with stable electronic properties.^28^ Among the various electrochemical techniques, impedimetric sensing has shown value despite the availability of several electrochemical techniques because of the advantages like wide response range, ability to detect the target analyte at low concentrations, better stability, high precision and sensitivity, small perturbation amplitude of from steady state, and great repeatability of the results.^29^

This communication is based on the preparation of a core–shell hybrid of electroactive functional PoPD with silver nanoparticles and the fabrication of the electrode to study enzyme-free electrochemical glucose sensing selectively. Here, the sensing performances and mechanisms are explained by considering the successful functionalization of silver metal nanoparticles through the functional groups of the polymer and successful adsorption on the surface of the electroactive functional polymer. Finally, the glucose content in the human blood sample was also verified by the designed sensor with the value of the standard commercial testing method.

## Experimental

### Materials

Crystalline synthesis grade *ortho*-phenylenediamine (Loba Chemicals Pvt. Ltd, India) was used as procured. The reagent grade chemicals, *viz.*, ammonium persulfate ((NH_4_)_2_S_2_O_8_, Merck, India), silver nitrate (AgNO_3,_ Merck, India), hydrochloric acid (HCl) (S. D. Fine Chemicals, India), sulfuric acid (H_2_SO_4_, Merck, India), DMSO (Merck, India), potassium chloride (KCl), potassium hexacyanoferrate (K_4_[Fe(CN)_6_]), and potassium ferricyanide (K_3_[Fe(CN)_6_]), were purchased and directly used as received. Deionized water with a resistance of 18 MΩ obtained from Milli-Q Plus (Millipore Inc.) water purifier was used for all purposes. The glassy carbon electrode (GCE) was purchased from Metrohm, Switzerland. Before use as a working electrode, it was cleaned entirely by rinsing, polishing and sonicating the carbon surface step-by-step as per the described procedure. The GCE was thoroughly washed with water, followed by polishing it with a pinch of alumina slurry on a soft cloth. To remove any residual contamination, the GCE was sonicated with de-ionized water in a beaker using an ultrasonic cleaner for 5 min. Then, the electrode was air-dried for an hour. The platinum counter electrode and standard Ag/AgCl reference electrode were also procured from Metrohm, Switzerland.

### Preparation of PoPD/Ag core–shell hybrid

The PoPD/Ag core–shell hybrid containing 7 wt% of silver was prepared as per the method reported recently.^30^ First, poly(*ortho*-phenylenediamine) containing free amine functional groups was synthesized in the aqueous sulfuric acid medium using ammonium persulfate (APS), as optimized earlier.^30^ In a typical method, a solution of monomer *o*-phenylenediamine (3.24 g, 30 mmol) was made in 45 mL aqueous H_2_SO_4_ with 1 : 1 acid-to-monomer mole ratio by constant stirring in the round-bottomed flask (RB) on a magnetic stirrer at room temperature. 30 mL aqueous solution of APS (10.26 g, 45 mmol) was added at once to the acid-monomer solution with constant magnetic stirring. The polymerization was carried out at room temperature under constant stirring for 10 h (minimum). The precipitate was collected by filtration and thoroughly washed with 2 M aqueous HCl solution to wash out the unreacted monomers, oligomers and impurities. Then, it was washed further several times with water until the light brown filtrate turned colourless and was dried well in an oven for 12 h at 70–80 °C. The PoPD/Ag core–shell hybrid was prepared in a single-step *in situ* process from the above synthesized polymer. In a two-necked scratch-free RB, a solution of 1 g PoPD and a calculated amount of AgNO_3_ (0.110 g) to obtain 7 wt% of silver was prepared in 20 mL DMSO with continuous stirring on a magnetic stirrer. The mixture was placed on a silicon oil bath and refluxed at 80 °C under a water condenser attached to one neck of the RB. The reaction was carried out in dark with the wrapping of black paper on the RB to avoid exposure to light for light-sensitive silver nitrate. After continuous refluxing at 80 °C for 2 h, a large volume (250 mL) of water was added to precipitate out the solid mass from the dense suspension. The solid residue was collected by vacuum filtration and washed with deionized water several times. Finally, the powder of the poly(*ortho*-phenylenediamine)/silver core–shell hybrid was dried at 60 °C for 10 h.

### Characterization

The structure of pristine PoPD and the PoPD/Ag hybrid was analysed using Fourier transform infrared (FTIR) spectroscopy with a Thermo Nicolet Nexus 870 FTIR. The IR absorption spectrum of the DMSO solution of the sample was obtained within the wavenumber range from 4000 to 500 cm^−1^ taking DMSO as the reference. The topography of the prepared hybrid powder was observed by both field emission scanning electron microscopy (FESEM) and high-resolution transmission electron microscopy (HRTEM) images. The FESEM image was recorded by taking the sample powder on a holder coated with carbon paper in a ZEISS SIGMA-300 FESEM instrument. Before scanning under the microscope for analysis, a thin platinum coating on the sample powder was applied by electro-sputtering. The HRTEM image of the sample powder was observed at an accelerated voltage of 200 kV in a JEOL JEM 2100 PLUS HRTEM instrument. Dynamic light scattering (DLS) analysis was performed to know the size distribution of the silver nanoparticles in the PoPD matrix. The characterization was carried out with a Zetasizer Nano Series Nano-ZS, Malvern using a very dilute solution of the PoPD/Ag hybrid in DMSO with respect to the reference of the DMSO solvent. For the analysis, one drop of the sample solution in DMSO was cast on a carbon-coated copper grid and dried at 50–60 °C overnight under vacuum. The ultraviolet visible (UV-vis) absorption spectrum of the very dilute aqueous suspension of the PoPD/Ag hybrid was recorded with a Micropack UV-vis-NIR DH 2000 spectrophotometer. The experiment was performed by taking the suspension in a quartz cuvette against a water reference in the range from 200 to 1000 nm. The UV-vis spectra were also recorded for the aqueous suspension of the PoPD/Ag hybrid after the addition of 4 and 10 μL aqueous solution of 72 mg per dL glucose solution. The Fourier transform infrared (FTIR) spectra of the PoPD/Ag hybrid with and without glucose were recorded using a Thermo Nicolet Nexus 870 FTIR. The sample for FTIR absorption spectrum was prepared by drying a drop of the alcoholic suspension of the sample on a glass slide between the wavenumber range of 4000 and 600 cm^−1^. The FTIR spectra were also recorded for the PoPD/Ag hybrid layer on a glass slide after drying 4 and 10 μL alcoholic solution of 72 mg per dL glucose.

### Fabrication of electrode and electrochemical study

**Fig. 1:**
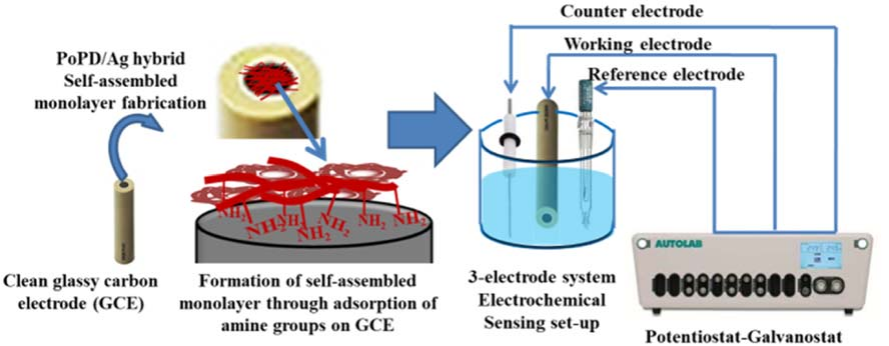
Illustration of the fabrication of GCE and three-electrode setup used to perform the electrochemical sensing measurement.

Electrochemical characterization was performed by recording CV and EIS in an AUTOLAB 302N potentiostat/galvanostat interfaced with a NOVA software. As shown in Fig. 1, a conventional three-electrode setup was used for electrochemical experiments. The three-electrode setup consisted of the as-fabricated GCE as the working electrode (Fig. 1), Ag/AgCl in KCl electrolyte as the reference electrode and platinum wire as the counter electrode. The self-assembled monolayer of the hybrid was cast on well-cleaned GCE to be used as a working electrode as follows. For the purpose, clean glassy carbon electrode was left in 0.5 mL DMSO solution of the 6–7 wt% hybrid overnight (10–12 h). Then, the electrode was thoroughly rinsed with water five/six times to remove DMSO or other residues and used directly as a working electrode for the electrochemical study. The CV and EIS were recorded in 25 mL of aqueous electrolyte containing 10^−3^ M [Fe(CN)_6_]^3−/4−^ redox couple at constant room temperature (25 °C ± 0.5 °C). The 10^−3^ M [Fe(CN)_6_]^3−/4−^ redox couple was prepared in 1 M aqueous KCl by dissolving a calculated amount of equimolar mixture of K_4_[Fe(CN)_6_] and K_3_[Fe(CN)_6_]. Potential scanning from −0.2 to +0.6 V was performed in CV measurements, and the EIS spectra were recorded within the range of frequency from 50 MHz to 100 kHz. To study the electrochemical sensing performances of modified GCE, the CV measurements were recorded in the same electrolyte under the same conditions after adding a particular concentration of aqueous glucose solution. Each time, 10 μL aqueous solution of glucose with respective concentrations, *viz.*, 72, 144, 216, 288, 360 mg dL^−1^ or 72 mg per dL glucose solution of 10, 20, 30, 40, 50 μL was added into 25 mL of the 1 M KCl electrolyte containing the 10^−3^ M [Fe(CN)_6_]^3−/4−^ redox couple. The selective response towards glucose over the interfering molecules like ascorbic acid, dopamine, uric acid, lactose, fructose, and sucrose was evaluated from chronoamperometric measurement by adding 100 μL aqueous solution of respective molecules. The response current of glucose influenced by the interfering species was evaluated by the sequential additions of 0.1 mM glucose or the above interfering biomolecules in the same electrolyte solution at an applied potential of 0.34 V Ag/AgCl. 10 and 50 μL of raw human blood were immediately dissolved in the same 25 mL electrolyte solution before coagulation and the solution was used for electrochemical analysis.

## Results and discussion

### Structure and morphology of the hybrid

**Fig. 2:**
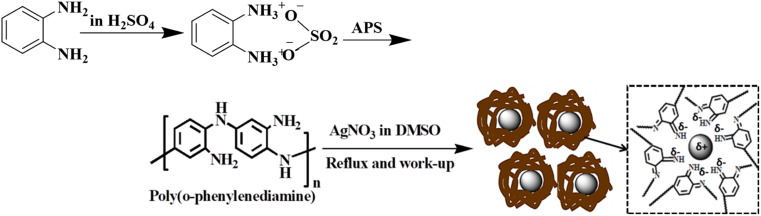
Synthesis of PoPD in aqueous medium using 1 : 1 sulfuric acid : monomer and the successive formation of the PoPD/Ag core–shell hybrid.

**Fig. 3:**
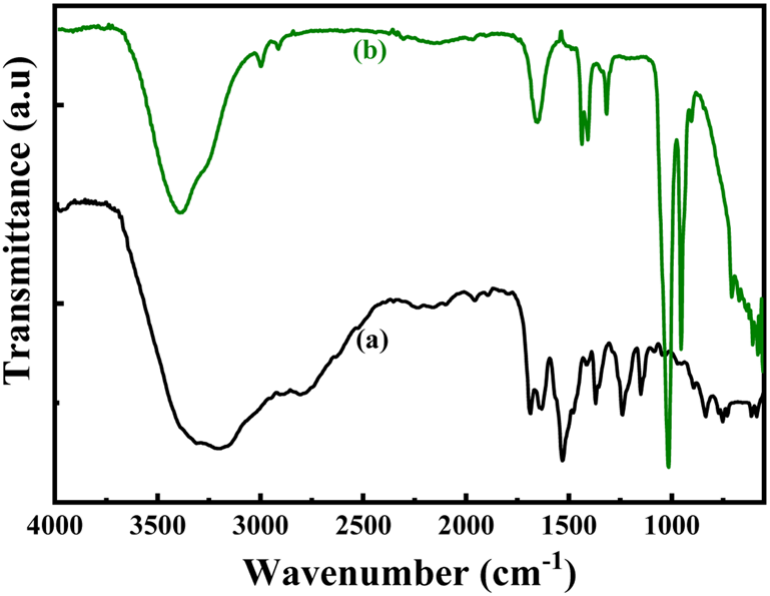
FTIR pattern of (a) pristine PoPD and (b) PoPD/Ag core–shell hybrid.

The structures of the pristine PoPD and the PoPD/Ag hybrid following the above optimized preparation method were briefly documented in our previous report.^27,28,30^ A scheme for the synthesis of PoPD and the successive formation of the core–shell hybrid with Ag is shown in Fig. 2.^27,28,30^ It was established that two –NH_2_ groups of one monomer were simultaneously protonated by single H_2_SO_4_ molecule in 1 : 1 acid : monomer polymerization medium (Fig. 2). Due to such strong protonation of both –NH_2_ groups of the monomer, the polymerization proceeds through only one –NH_2_ groups to result in a structure like polyaniline derivative with free –NH_2_ functional groups.^27,28,30^ The structure of the pristine PoPD and PoPD/Ag hybrid was further confirmed from the FTIR spectral analysis (Fig. 3). As reported earlier,^27,28,30^ the common bands appeared in the FTIR spectrum of pristine PoPD; at 3690 cm^−1^ for aromatic C–H stretching, at 2250 cm^−1^ for the stretching of hydrogen bonded –NH_2_ and/or <svg xmlns="http://www.w3.org/2000/svg" version="1.0" width="10.400000pt" height="16.000000pt" viewBox="0 0 10.400000 16.000000" preserveAspectRatio="xMidYMid meet"><metadata> Created by potrace 1.16, written by Peter Selinger 2001-2019 </metadata><g transform="translate(1.000000,15.000000) scale(0.011667,-0.011667)" fill="currentColor" stroke="none"><path d="M80 1160 l0 -40 40 0 40 0 0 -40 0 -40 40 0 40 0 0 -40 0 -40 40 0 40 0 0 -40 0 -40 40 0 40 0 0 -40 0 -40 40 0 40 0 0 -40 0 -40 40 0 40 0 0 -40 0 -40 40 0 40 0 0 80 0 80 -40 0 -40 0 0 40 0 40 -40 0 -40 0 0 40 0 40 -40 0 -40 0 0 40 0 40 -40 0 -40 0 0 40 0 40 -40 0 -40 0 0 40 0 40 -80 0 -80 0 0 -40z M560 520 l0 -40 -40 0 -40 0 0 -40 0 -40 -40 0 -40 0 0 -40 0 -40 -40 0 -40 0 0 -40 0 -40 -40 0 -40 0 0 -40 0 -40 -40 0 -40 0 0 -40 0 -40 -40 0 -40 0 0 -40 0 -40 80 0 80 0 0 40 0 40 40 0 40 0 0 40 0 40 40 0 40 0 0 40 0 40 40 0 40 0 0 40 0 40 40 0 40 0 0 40 0 40 40 0 40 0 0 80 0 80 -40 0 -40 0 0 -40z"/></g></svg> NH– groups in the polymer chain, at 1615 cm^−1^ for the stretching of quinoid C–C, including the deformation of N–H groups, 1500 cm^−1^ for the stretching of benzenoid C–C, 1373 cm^−1^ for the stretching of quinoid C–N and 1243 cm^−1^ for the stretching of benzenoid C–N (Fig. 3). The presence of free amine functional groups in the pristine PoPD was confirmed from the shoulder peak of C–N symmetric stretching at 1362 cm^−1^ including two bending vibrational bands of the –NH_2_ groups at 1685 cm^−1^ and 1631 cm^−1^ (Fig. 3). In the FTIR spectrum of the PoPD/Ag hybrid, the stretching bands of aromatic C–H, –NH_2_ and/or NH– groups were shifted towards a higher wavenumber, and all the above other bands were shifted towards a lower wavenumber compared to the pristine PoPD due to strong interfacial electrostatic interaction with the silver nanoparticles.^28^ The characteristic bending vibrational bands of the –NH_2_ groups at 1685 cm^−1^ and 1631 cm^−1^ was combined to a single broad band at 1622 cm^−1^ corresponding to the stretching of C=N (Fig. 3). As reported,^28^ a well-defined core–shell structure of the PoPD/Ag hybrid was formed with 0.124 g g^−1^ or ∼12% silver loading. Uniformly distributed Ag(0) nanoparticles were formed by the reduction of Ag^+^ ion within the polymer–amine complex, followed by the simultaneous oxidation of –NH_2_ to =NH groups in the PoPD. The stability and electroactivity of the core–shell hybrid was explained by considering the strong interfacial electrostatic interaction of the electropositive silver nanoparticles core with the electronegative =NH groups of the PoPD shell (Fig. 2).

**Fig. 4:**
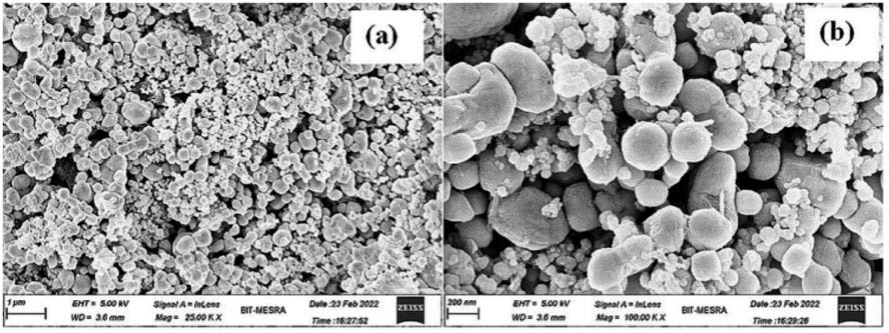
FESEM of the PoPD/Ag core–shell hybrid at (a) 25 k× magnification and (b) 100 k× magnification.

**Fig. 5:**
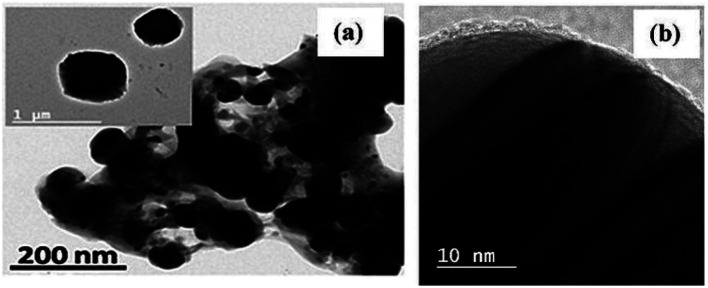
HRTEM of (a) the PoPD/Ag core–shell hybrid with two individual core–shell structures shown in the inset and (b) the enlarged view of the PoPD shell coating on the surface of the Ag core.

As shown in Fig. 4 and 5, the core–shell formation was confirmed from the respective FESEM and HRTEM images of the hybrid. The formation of spherical beads having diameter ranging from 100 to 300 nm with an average of 140 nm was noted within a wide region of the FESEM image (Fig. 4a). In the enlarged view, the definite core–shell structure was formed by the uniform wrapping/coating of the PoPD shell on the core of the silver surface (Fig. 4b). Some bulk morphology was also identified in Fig. 4 due to the combination of a few spherical core–shell structures together. The FESEM result was correlated with the HRTEM images of the core–shell hybrids shown in Fig. 5. Here, the average diameter of spherical beads was noted as 160 nm ranging from 20 to 400 nm, including some bulk morphology as well (Fig. 5a). As reported earlier,^28^ the thickness of 5–50 nm was calculated for the PoPD shell on the silver core, having a uniform diameter of 85–97 nm. In addition, the size distribution of the silver core was also confirmed from the particle size histogram analysis by DLS characterization (Fig. S1†). Very uniform spherical silver nanoparticles were distributed within the diameter range of 85–98 nm in the histogram (Fig. S1†). Moreover, 100% of the nanoparticles were found to have an average diameter of 91.28 nm within this narrow range of size distribution (Fig. S1†). A large core–shell sphere with a diameter of 400 nm and another average sphere with a diameter of 160 nm is shown in the inset view of Fig. 5a. As shown in Fig. 5b, a uniform wrapping/coating of 2–8 nm average thickness of the PoPD with black colour was noticeable on the surface of the light-coloured silver sphere. It should also be noted that after PoPD/Ag hybrid formation, the terminal –NH_2_ groups and some –NH_2_ groups as ring substituents would be free.^28^ As shown in Fig. 1, those free –NH_2_ groups were adsorbed on the glassy carbon electrode surface and formed the self-assembled layer of the PoPD/Ag hybrid.^13,31,32^

### Electrochemical properties of the modified electrode

**Fig. 6:**
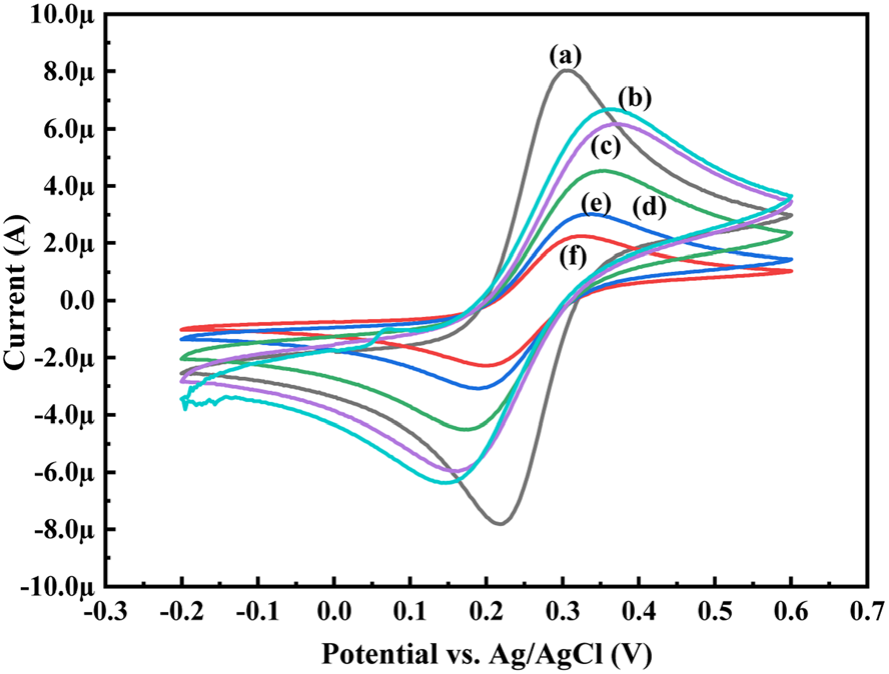
Comparative CV of (a) GCE at scan rate 50 mV s^−1^, (b) PoPD-modified GCE at a scan rate of 50 mV s^−1^, (c) PoPD/Ag-modified GCE at a scan rate of 50 mV s^−1^, (d) PoPD/Ag-modified GCE at a scan of rate 25 mV s^−1^, (e) PoPD/Ag-modified GCE at a scan rate of 10 mV s^−1^, and (f) PoPD/Ag-modified GCE at a scan rate of 5 mV s^−1^ in the aqueous KCl (1 M) electrolyte containing the 10^−3^ M [Fe(CN)_6_]^3−/4−^redox couple.

**Fig. 7:**
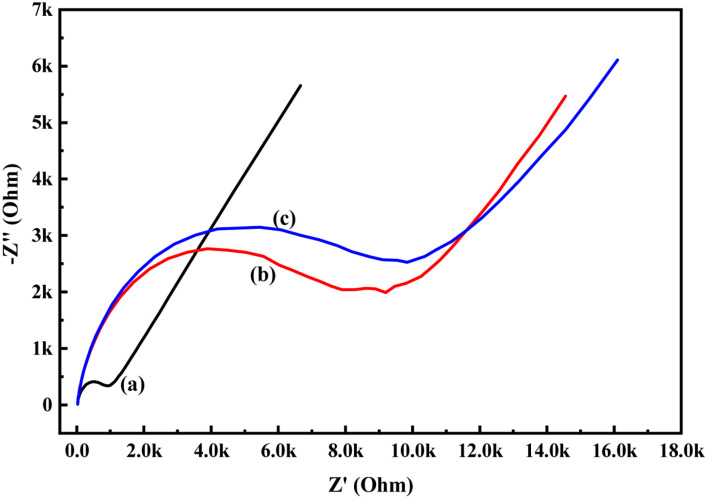
Comparative EIS of (a) GCE, (b) PoPD/Ag-modified GCE, (c) PoPD-modified GCE in the aqueous KCl (1 M) electrolyte containing the 10^−3^ M [Fe(CN)_6_]^3−/4−^redox couple.

In order to know the electrochemical properties of the PoPD/Ag hybrid layer self-assembled on the GCE electrode, CV and EIS were performed in the aqueous KCl electrolyte containing the [Fe(CN)_6_]^3−/4−^ redox couple. The comparative CV and EIS of the different electrodes are shown in Fig. 6 and 7. A perfect sigmoidal and reversible CV curve having significantly lower peak current was observed for PoPD/Ag-coated GCE than that of the bare GCE (Fig. 6). However, the peak current appeared to increase than that of the pristine PoPD self-assembled on the GCE electrode. Upon coating with PoPD/Ag, the easy interfacial diffusion of electrons through the PoPD layer mediated by the silver nanoparticles might be considered to correlate with the nature of the curve. The Δ*E* value (separation of anodic and cathodic peak current) of ∼239 mV was noted for PoPD or PoPD/Ag self-assembled GCE electrode with respect to ∼93 mV for the bare GCE (Fig. 6). It is apparent that the electron transfer during the potential scan was considered poor for the PoPD or PoPD/Ag electrode due to the self-assembly of the poorly conducting polymer layer. The influence of variable scan rates on the CV pattern of the PoPD/Ag electrode is also presented in Fig. 6. It was noticed that the anodic peak, cathodic peak and Δ*E* value of the PoPD/Ag electrode was regularly increased with increasing scan rate (Fig. 6). It can be concluded that the self-assembled layer of PoPD/Ag on GCE offers sufficient electrochemical activity for good electron transport between the surface of the electrode and the medium of the electrolyte. The same inference might be drawn from the EIS measurements of those electrodes (Fig. 7). A Nyquist diagram of imaginary value (−*Z*′′) against the real value (*Z*′) was obtained by considering the capacitive and resistive-controlled process for such a system containing an electrode/electrolyte interface. Using the NOVA software, the corresponding equivalent circuit was fitted with the elements; the ohmic resistance of the solution electrolyte (*R*), resistance for charge transfer at the interface (*R*_ct_), and Warburg element including constant phase element (CPE). In the Nyquist plot, the semicircle portion indicates the value of the electron transfer rate (*R*_ct_) corresponding to the limited electron transfer process at the electrode/electrolyte interface. As determined, the clean GCE was found to have an *R*_ct_ value of 1.5 kΩ, which was altered to 10.25 kΩ for the PoPD/Ag electrode and 12.49 kΩ for the PoPD electrode (Fig. 7). Hence, the PoPD/Ag hybrid self-assembled electrode was found to have better electron transfer capability over the pristine PoPD layer due to the presence of conductive silver nanoparticles. The electrode PoPD/Ag electrode has sufficiently stable electrochemical performance for successful use as an electrochemical sensing layer.

### Electrochemical sensing performance

**Fig. 8:**
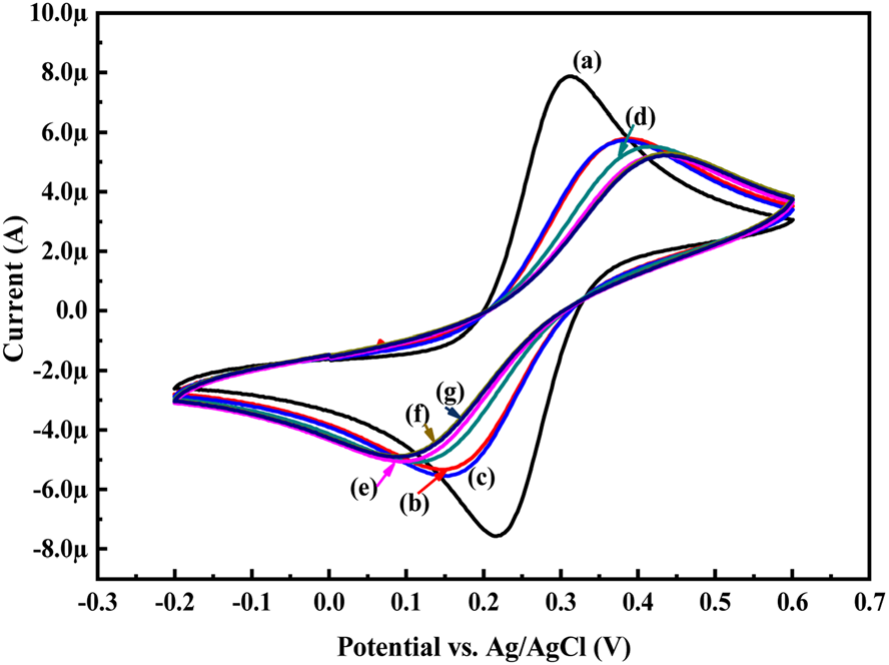
CV responses of (a) GCE, (b) PoPD/Ag hybrid-modified GCE and PoPD/Ag hybrid-modified GCE towards (a) 10, (b) 20, (c) 30, (d) 40, and (e) 50 μL of 72 mg per dL aqueous glucose at a scan rates of 50 mV s^−1^ in 25 mL aqueous KCl (1 M) electrolytes containing the 10^−3^ M [Fe(CN)_6_]^3−/4−^redox couple.

**Fig. 9:**
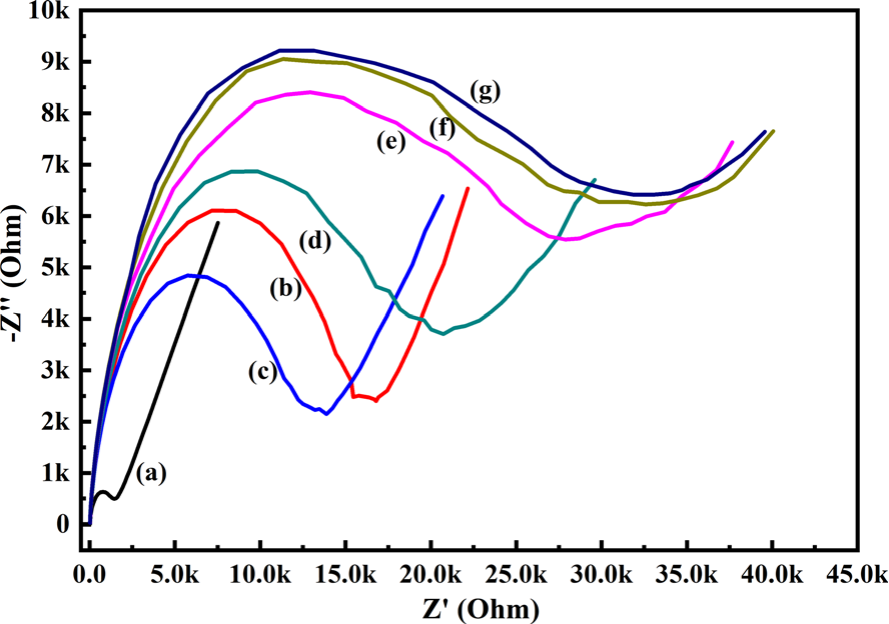
EIS responses of (a) GCE, (b) PoPD/Ag hybrid-modified GCE and PoPD/Ag hybrid-modified GCE towards (c) 10, (d) 20, (e) 30, (f) 40, and (g) 50 μL of 72 mg per dL aqueous glucose in 25 mL aqueous KCl (1 M) electrolytes containing the 10^−3^ M [Fe(CN)_6_]^3−/4−^ redox couple.

To evaluate the electrochemical activity towards glucose, the CV and EIS analysis of the PoPD/Ag hybrid layer self-assembled on the GCE electrode was done in the presence of glucose. Fig. 6 shows the CV patterns of the PoPD/Ag hybrid-modified GCE at varying concentrations of 10 μL glucose at a constant scan rate of 50 mV s^−1^. As shown in Fig. 8, a slight increase in the anodic peak current from 5.40 μA to 5.53 μA was observed for the PoPD/Ag electrode upon the addition of 10 μL of 72 mg per dL glucose (Table S1†). Then, both anodic and cathodic peak current were gradually decreased (Table S1†) with increasing concentration by the further addition of glucose (Fig. 8). The CV profiles of the PoPD/Ag hybrid modified GCE in the presence of 20 μL of 72 mg per dL glucose at varying scan rates ranging from 5 to 100 mV s^−1^ are shown in Fig. S2.† As shown in Fig. S2,† a gradual increase in the anodic and cathodic peak current was recorded for a constant glucose concentration with increasing scan rates. A good linear relationship with a correlation coefficient (*R*-square) value of 0.93 was fitted for the plot of anodic peak current *vs.* scan rate (Fig. S3†). This implies the absorption-controlled mechanism of glucose on the PoPD/Ag hybrid layer. Similar to that of CV analysis, the sensing of glucose by the PoPD/Ag hybrid-modified GCE was also performed using EIS analysis (Fig. 9). As shown in Fig. 9, the *R*_ct_ value of the PoPD/Ag electrode was slightly decreased with the first addition of 10 μL of 72 mg per dL glucose (Table S2†). Then, the *R*_ct_ value of the PoPD/Ag electrode was gradually increased upon the successive addition of glucose (Table S2†). Hence, the electron transfer process at the electrode/electrolyte interface through the PoPD/Ag hybrid layer was restricted by the increasing surface adsorption of glucose except for the first step of adsorption. In contrast, a negligible increase in the anodic peak current was also recorded in the CV sensing response of the pristine PoPD electrode towards 10 μL of 72 mg per dL glucose (Fig. S4†). However, no significant change of the *R*_ct_ value of the PoPD/Ag electrode in EIS response was observed for the sensing of 10 μL of 72 mg per dL glucose (Fig. S5†). Therefore, it can be said that pristine PoPD was not found to be suitable for the sensing of glucose. This is because of the poor electrochemical activity of pristine PoPD without silver nanoparticles and the lack of =NH functionality for significant interactions with glucose. Here, the role of silver nanoparticles is to increase the electrochemical activity of the layer by the strong electrostatic doping effect on the PoPD matrix and to promote the electrochemical process through easy electron transfer.

### Proposed sensing mechanism

**Fig. 10:**
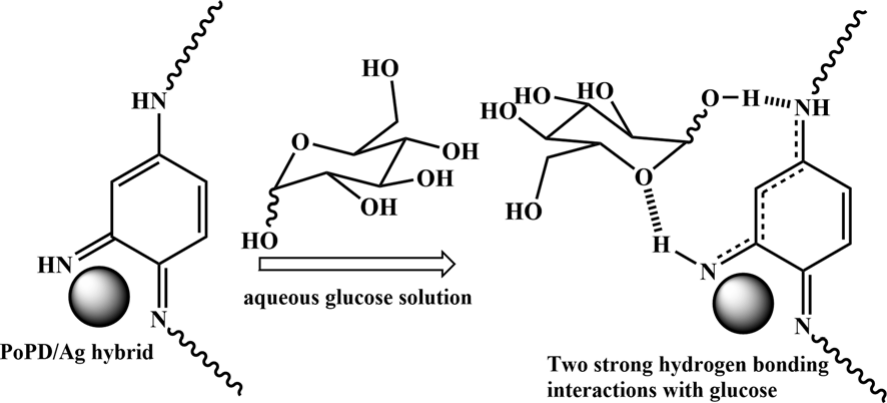
Glucose sensing mechanism on PoPD/Ag-modified GCE electrode.

**Fig. 11:**
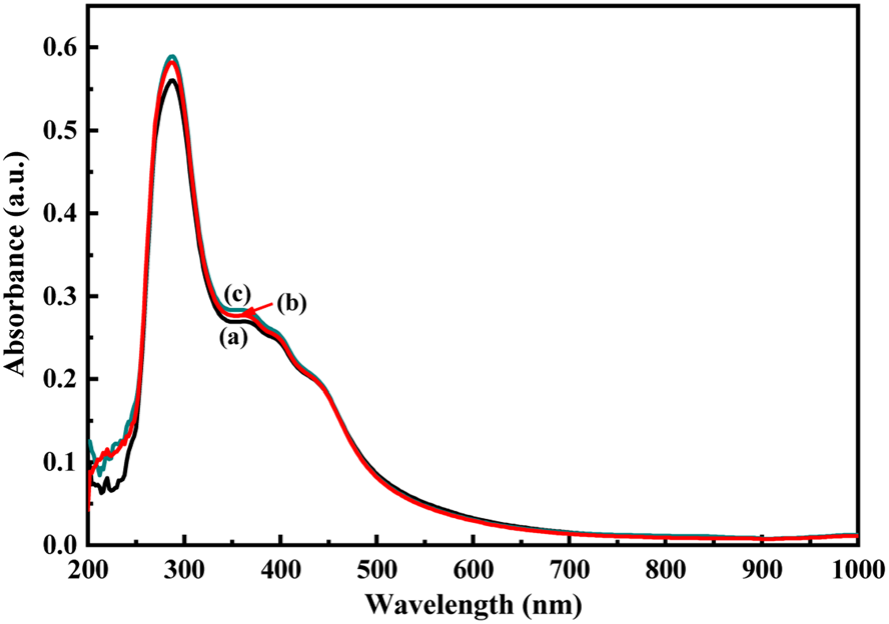
UV-vis spectra of the aqueous suspension of (a) the PoPD/Ag hybrid and with the addition of (b) 4 and (c) 10 μL of glucose.

**Fig. 12:**
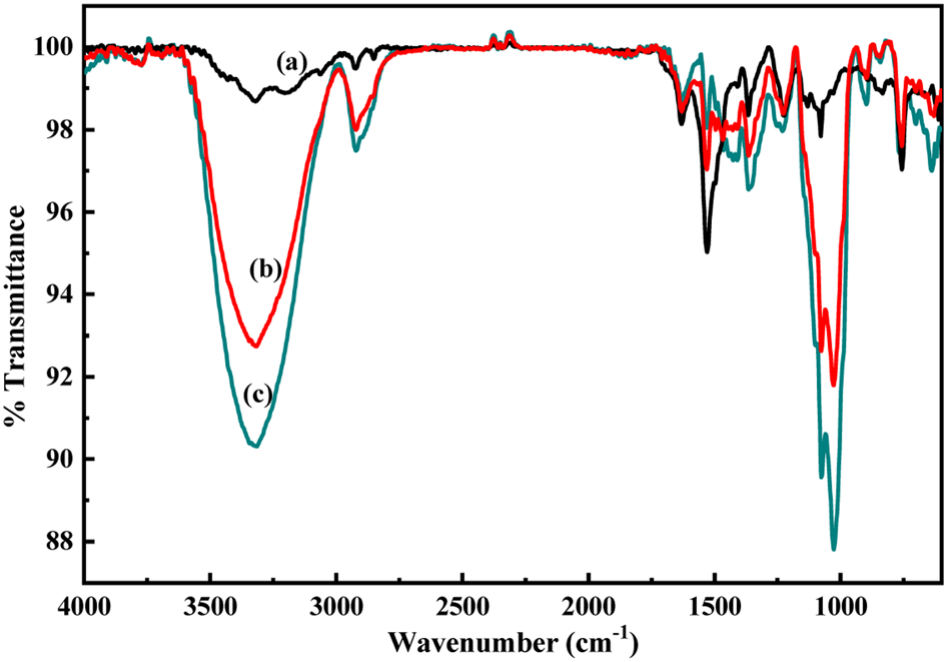
FTIR spectra of the aqueous suspension of (a) the PoPD/Ag hybrid and with the addition of (b) 4 and (c) 10 μL of glucose.

As described in the previous section, the first step of glucose interaction with PoPD/Ag on the GCE produced different CV or EIS responses than that of the successive steps. A slight increase in the peak current and a decrease in the charge transfer resistance was observed for the first addition of glucose in the electrolyte solution. This means that the electron transfer process at the electrode/electrolyte interface through the PoPD/Ag hybrid layer was promoted at the first step surface adsorption of glucose. This can be correlated with the increasing hydrophilicity of the PoPD/Ag hybrid layer through the strong hydrogen bonding interaction with glucose in the pyranose form (Fig. 10). For evidence, an increase in the peak intensity was observed in the UV-vis spectra of the PoPD/Ag hybrid in the presence of glucose without altering the normal peak position (Fig. 11). As reported earlier,^18^ the UV-vis peaks appeared at about 317 nm for π–π* transition, 475 nm for excitonic transition, 395 nm for polaron electron transition of Ag nanoparticle-doped PoPD, and 405 nm for surface plasmon resonance absorption band of the silver nanoparticles (Fig. 11). The shifting of peak position was reordered compared to the earlier report due to the poor solvation or insolubility of the PoPD/Ag hybrid within the used water solvent.^21^ Here, two types of hydrogen bonding interactions were explained: the =N–H groups of the PoPD in nanocomposite with the pyranose C–O–C of glucose and the –NH– groups of PoPD in the nanocomposite with the C1–OH of glucose. The particular size and shape of the glucopyranose is considered to be responsible for such type of strong interactions. However, possibilities of hydrogen bonding with other –OH groups might not be ruled out. For the hydrogen bonding, the quinoid rings were delocalized to the benzenoid structure (Fig. 10). In FTIR, the intensity of the quinoid C–N stretching band at 1373 cm^−1^ was increased and that of the benzenoid C–N stretching band at 1243 cm^−1^ was decreased (Fig. 12). Moreover, the intensity of the band at 1615 cm^−1^ for the =N–H deformation of the secondary amine was significantly decreased with the increasing concentration of glucose (Fig. 10). As shown in Fig. 10, the structure of the PoPD in the PoPD/Ag hybrid is important to have two simultaneous strong hydrogen bonding interactions. However, this type of hydrogen bonding interaction is absent due to the absence of the =NH functionality in the pristine PoPD and hence it did not produce response with glucose (Fig. 10). With the further addition of glucose, the hydrophilicity was increased very little as the UV-vis peak intensity was increased a little (Fig. 11). At higher concentration, the increase in the electrochemical inactive glucose adsorption on the PoPD/Ag hybrid through hydrogen bonding limited the transfer of electron at the electrode/electrolyte interface. Thus, the peak current was decreased but the charge transfer resistance was increased upon the successive addition of glucose.

### Analysis of sensing parameters

**Fig. 13:**
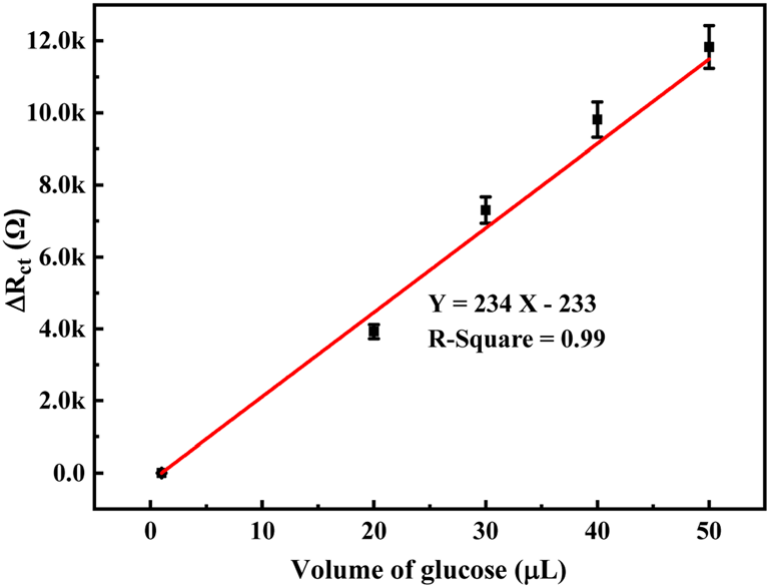
Calibration plot of the change in difference of *R*_ct_ in the presence of glucose analyte with that of the PoPD/Ag electrode against the concentration of glucose.

**Fig. 14:**
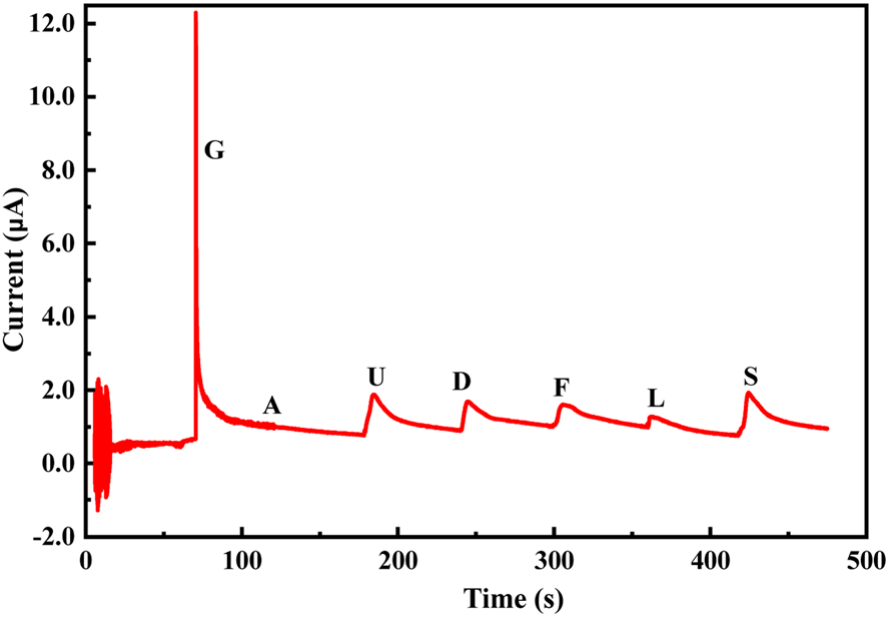
Chronoamperometric response of the PoPD/Ag hybrid-modified GCE towards 10 μL of 0.1 mM glucose (G), ascorbic acid (A), uric acid (U), dopamine (D), fructose (F), lactose (L) and sucrose (S) in 25 mL aqueous KCl (1 M) electrolytes containing the 10^−3^ M [Fe(CN)_6_]^3−/4−^redox couple.

The change in the anodic and cathodic peak current against the concentration of glucose is shown in Fig. S6.† A very poor linear relationship of correlating the coefficient value (*R*-square) of 0.67–0.71 was obtained for both the above trends (Fig. S6†). Therefore, it is very hard to find any good reliable result from the sensing response by CV. The change in the *R*_ct_ of the PoPD/Ag electrode in the EIS experiment against the concentration of glucose is shown in Fig. S7.† A very good linear fitting was obtained, having a correlating coefficient value (*R*-square) of 0.988. However, the change in the difference of *R*_ct_ in the presence of glucose analyte with that of the PoPD/Ag electrode against the concentration of glucose was found to be appropriate in terms of sensing (Fig. 13). This is because the GCE may not always have the same value after the self-assembly of the PoPD/Ag hybrid. An ideal linear fitting of the plot was drawn, having a correlating coefficient value (*R*-square) of 0.99 with the increasing concentration of glucose (Fig. 13). The sensor exhibited a sensitivity of 298 Ω mg^−1^ dL cm^−2^, computed from equation:^33^ sensitivity = slope of the line/active surface area of the GCE electrode (0.785 cm^2^) (Fig. 13). This sensitivity was found to be superior over similar types of impedimetric sensors.^10–17^ An experimental limit of detection (LOD) or limit of quantification (LOQ) for the sensor was determined to be 10 μL of ∼80 mg per dL glucose solution in 25 mL of 1 (M) KCl electrolyte containing the 10^−3^ M [Fe(CN)_6_]^3−/4−^ redox couple. The *R*_ct_ value from EIS analysis was found to decrease for a glucose concentration lower than the LOD value. However, the sensing of lower glucose concentration than ∼80 mg dL^−1^ may also be possible using a relatively high volume of the sample. The PoPD/Ag sensor also showed the excellent selectivity for glucose over the other interfering biomolecules. The selectivity and interference of glucose sensing response were studied with interfering molecules like ascorbic acid (A), uric acid (U), dopamine (D), fructose (F), lactose (L) and sucrose (S). The amperometric response of the PoPD/Ag hybrid-modified GCE upon the addition of glucose and other interfering compounds is shown in Fig. 14. Upon the addition of glucose, the current response instantly increased and immediately dropped down to its original position (Fig. 14). This is due to the increase in the π-electron density by the instant conversion of the quinoid to the delocalized benzenoid structure through the hydrogen bonding interaction of glucose molecules on the surface of the PoPD/Ag layer.^34^ At the moment of interaction, the dipolar nature of the PoPD/Ag layer was increased immediately to produce a current response (Fig. 14). From the plot, it was also said that the sensor was found to have a very small (>1 s) response time. However, a very poor signal appeared for the successive addition of the above interfering biomolecules, implying the very weak hydrogen bonding interactions of those molecules with the PoPD/Ag hybrid on GCE. As mentioned, the structural difference of those molecules, including their size and shape, with that of glucopyranose was considered the driving force for weak hydrogen bonding interactions of these molecules.

### Raw human blood sample analysis

**Fig. 15:**
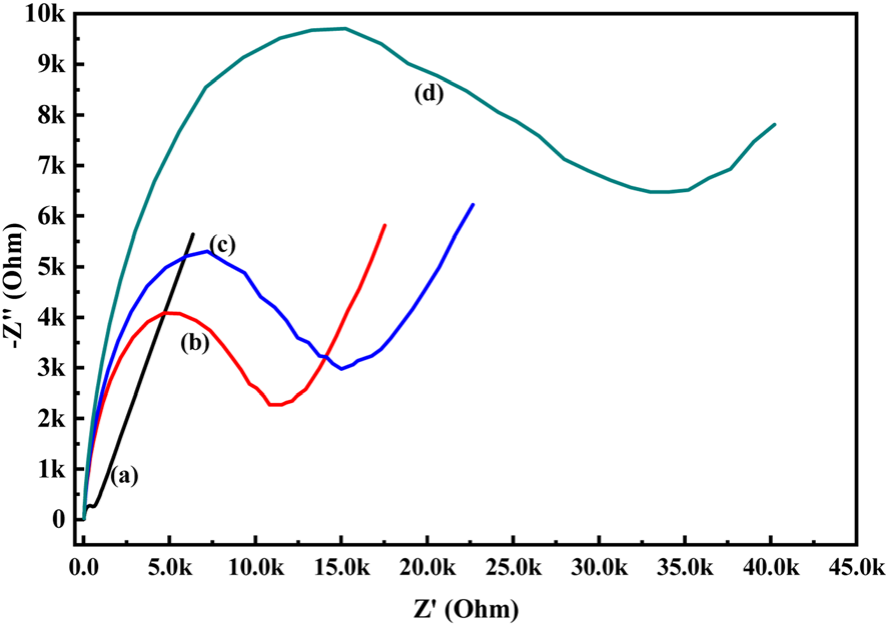
EIS responses of (a) GCE, (b) PoPD/Ag hybrid-modified GCE towards and PoPD/Ag hybrid-modified GCE towards (c) 10 μL, (d) 50 μL blood sample in 25 mL aqueous KCl (1 M) electrolytes containing the 10^−3^ M [Fe(CN)_6_]^3−/4−^ redox couple.

To verify the present method of analysis, the glucose levels in the blood samples were evaluated using the calibration curve obtained in this method and was compared with the result obtained from the commercial kit (Dr Morepen BG-03 Gluco One Glucometer). The CV responses of the PoPD/Ag hybrid-modified GCE towards 10 μL and 50 μL raw blood samples in 25 mL aqueous KCl (1 M) electrolytes containing the 10^−3^ M [Fe(CN)_6_]^3−/4−^redox couple is shown in Fig. S8.† As expected, a decreasing trend of both anodic and cathodic peak currents was recorded by the addition of 10 μL and 50 μL raw blood samples (Table S3†). The sensing result of the raw blood sample by the PoPD/Ag hybrid-modified GCE was also performed from EIS analysis (Fig. 15). An increasing trend of the *R*_ct_ value of the PoPD/Ag electrode was recorded by increasing the volume of raw blood from 10 to 50 μL (Table S4†). The glucose concentration in the blood sample was calculated using the following equation derived from the above calibration plot (Fig. 13).
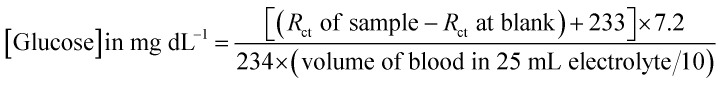
Here, 7.2 was obtained for 1 μL solution from 10 μL of 72 mg per dL glucose solution in 25 mL electrolyte. The values 233 and 234 are the intercept and slope of the linear fitted calibration plot, respectively (Fig. 13). In case of different sample volumes, it should be expressed in terms of 10 μL of 72 mg per dL glucose solution by dividing with 10. The raw blood sample analysis result is included in Table 1. As shown in Table 1, the glucose level of raw blood was calculated using the above equation as 97–101 mg dL^−1^ against the glucose level of 100 mg dL^−1^ obtained by the commercial kit (Dr Morepen BG-03 Gluco One Glucometer). Hence, the result is very close with 97–101% recovery or accuracy with the present method.

**Table 1:** Raw human blood sample analysis result

Volume of blood sample (μl)	*R*_ct_ (Ω)	Δ*R*_ct_ with blank measurement (Ω)	Calculated [glucose] in blood sample (mg dL^−1^)	% recovery or accuracy
0 (blank)	10 452	—	—	—
10	13 379	2927	97.2 ± 1.6	97
50	26 685	16 233	101.3 ± 0.5	101

## Conclusions

A promising electrochemical technique has been demonstrated for the successful enzyme-free impedimetric sensing of glucose using the PoPD/Ag core–shell hybrid-modified GCE. The PoPD/Ag hybrid self-assembled layer on the GCE electrode was found to have excellent stable electrochemical properties for use as an impedimetric sensing probe. Very good impedimetric sensing response was observed over the voltametric response towards glucose in the aqueous electrolyte containing the [Fe(CN)_6_]^3−/4−^ redox couple. As explained, the charge transfer resistance of the PoPD/Ag core–shell hybrid-modified GCE was decreased with the increasing concentration of glucose in the electrolyte. A very good linear fitting calibration plot was noted for charge transfer resistance against the varying volume of a particular concentration of glucose. The sensitivity of 298 Ω mg^−1^ dL cm^−2^ was calculated for the PoPD/Ag core–shell hybrid-modified GCE-based sensor with a detection limit of 10 μL of ∼80 mg per dL glucose solution in 25 mL of 1 M KCl electrolyte containing the 10^−3^ M [Fe(CN)_6_]^3−/4−^ redox couple. The sensing response of the PoPD/Ag core–shell hybrid-modified GCE towards glucose was found to be selective over the interfering molecules like ascorbic acid, uric acid, dopamine, fructose, lactose and sucrose. As verified, the glucose level of raw human blood was calculated as 97–101 mg dL^−1^, having almost 100% recovery or accuracy against the glucose level of 100 mg dL^−1^ obtained by the commercial kit (Dr Morepen BG-03 Gluco One Glucometer).

## Data availability

The data will be made available on request.

## Author contributions

Mukul Deo: formal analysis, data curation, methodology, investigation, writing–review & editing. Devleena Sahoo: data curation, methodology, writing–original draft. Pradip Kar: conceptualization, methodology, supervision, validation, writing– review & editing.

## Conflicts of interest

The authors declare that they have no known competing financial interests or personal relationships that could have appeared to influence the work reported in this paper.

## Supplementary Material

RA-014-D4RA04766D-s001
