# Supplementary material for: Self-assembled monolayer of poly(o-phenylenediamine)/silver core–shell hybrid-based enzyme-free impedimetric glucose sensor for blood samples
Source: RSC Adv. 2024 Aug 27;14(37):26863–72. doi: 10.1039/d4ra04766d (PMC11347925; doi:10.1039/d4ra04766d)
Supplement: RA-014-D4RA04766D-s001 [file RA-014-D4RA04766D-s001.pdf]

Supplementary Information (SI) for RSC Advances.
This journal is © The Royal Society of Chemistry 2024

Self-assembled monolayer of poly(o-phenylenediamine)/silver core-shell hybrid based enzyme-free impedimetric glucose sensor for blood sample

Mukul Deo[a], Devleena Sahoo[a], Pradip Kar†[a]

[a] Department of Chemistry, Birla Institute of Technology, Mesra, Ranchi-835215, Jharkhand, India

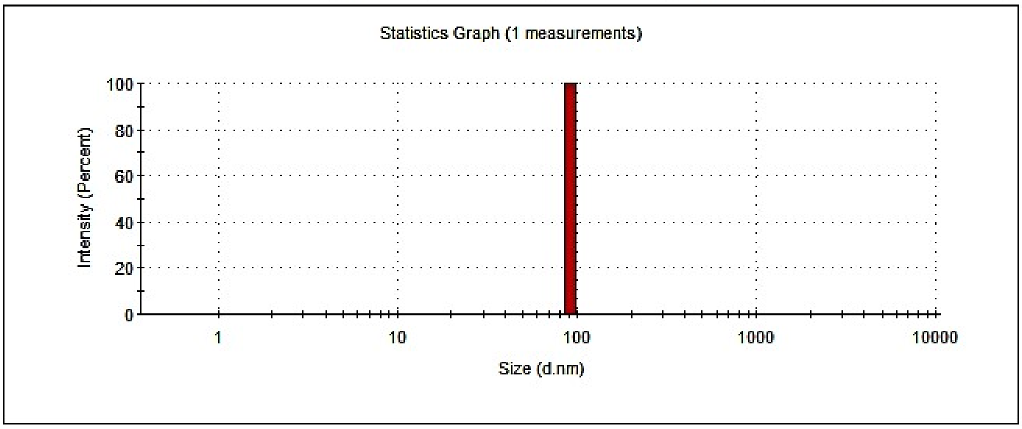

Fig S1. Size distribution of Ag nanoparticles within PoPD matrix.

Table S1. Anodic peak current (I_{pa}), cathodic peak current (I_{pc}), Anodic peak potential (E_{pa}), cathodic peak potential (I_{pc}) and potential difference (ΔE) for the CV responses of glucose analyte

Sl. no.	Specification	I_{pa} (μA)	I_{pc}(μA)	E_{pa}(V)	E_{pc}(V)	ΔE= E_{pa} -E_{pc} (mV)
01	Clean GCE	7.859	7.565	0.310	0.217	93
02	PoPD/Ag hybrid modified GCE	5.780	5.327	0.386	0.150	239
03	PoPD/Ag hybrid modified GCE towards 10 μL of 72 mg dL^{-1} glucose	5.715	5.546	0.384	0.151	233
04	PoPD/Ag hybrid modified GCE towards 20 μL of 72 mg dL^{-1} glucose	5.518	5.081	0.415	0.115	300
05	PoPD/Ag hybrid modified GCE towards 30 μL of 72 mg dL^{-1} glucose	5.229	5.071	0.426	0.097	329
06	PoPD/Ag hybrid modified GCE towards 40 μL of 72 mg dL^{-1} glucose	5.290	4.885	0.435	0.088	352
07	PoPD/Ag hybrid modified GCE towards 50 μL of 72 mg dL^{-1} glucose	5.221	4.899	0.437	0.087	351

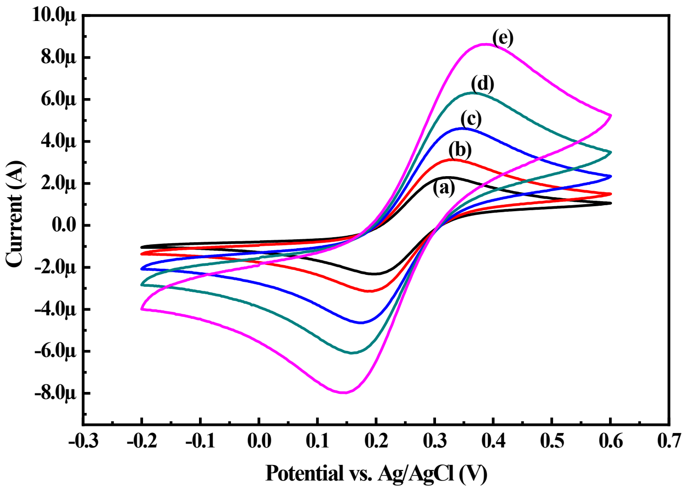

Fig. S2. Comparative CV of PoPD/Ag modified GCE towards 10 µL of 72 mg dL^{-1} glucose in 25 mL aqueous KCl (1 M) electrolytes containing 10^{-3} M $[Fe(CN)_6]^{3-/4-}$ at scan rate (a) 5, (b) 10, (c) 25, (d) 50 and (e) 100 mV s^{-1}

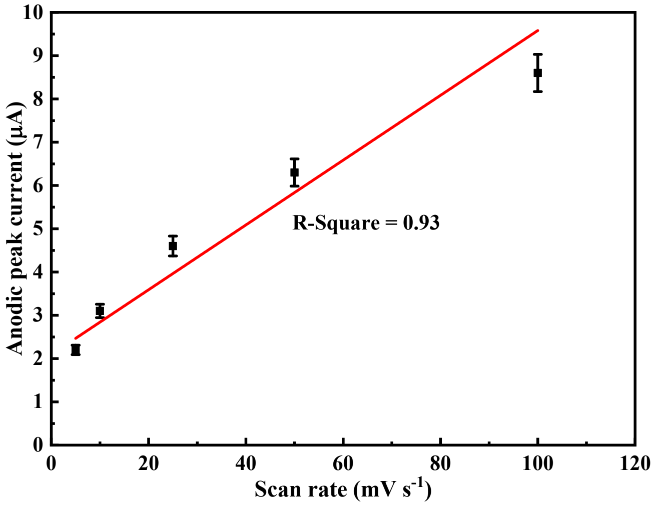

Fig. S3. Linear fitting plot of the anodic peak current against the scan rate

Table S2. Fitted Rct values and different of Rct in EIS measurements of glucose analyte

Sl. No.	Specification	R_{ct} (Ω)	+/-(Ω)	different of Rct with blank (Ω)
01	Clean GCE	1535	5	
02	PoPD/Ag hybrid modified GCE	15800	100	0
03	PoPD/Ag hybrid modified GCE towards 10 µL of 72 mg dL^{-1} glucose	12325	50	-3475
04	PoPD/Ag hybrid modified GCE towards 20 µL of 72 mg dL^{-1} glucose	19720	150	3920
05	PoPD/Ag hybrid modified GCE towards 30 µL of 72 mg dL^{-1} glucose	23105	105	7305
06	PoPD/Ag hybrid modified GCE towards 40 µL of 72 mg dL^{-1} glucose	25610	120	9810
07	PoPD/Ag hybrid modified GCE towards 50 µL of 72 mg dL^{-1} glucose	27630	250	11830

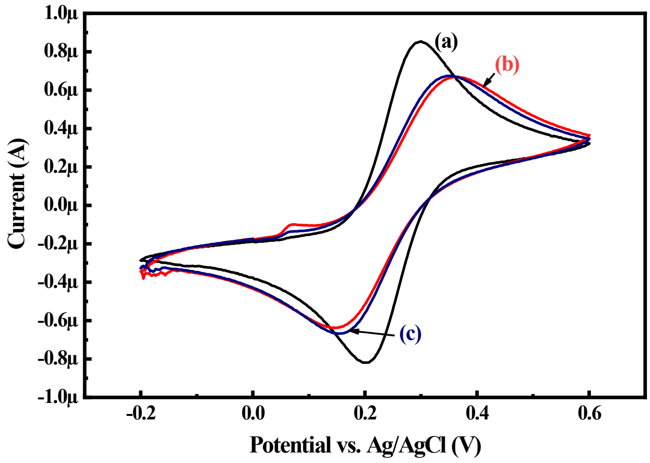

Fig. S4. CV responses of (a) GCE, (b) PoPD modified GCE and (c) PoPD modified GCE towards 10 μL of 72 mg dL^{-1} aqueous glucose at scan rate 50 mV s^{-1} in 25 mL aqueous KCl (1 M) electrolytes containing 10^{-3} M $[Fe(CN)_6]^{3-/4-}$redox couple

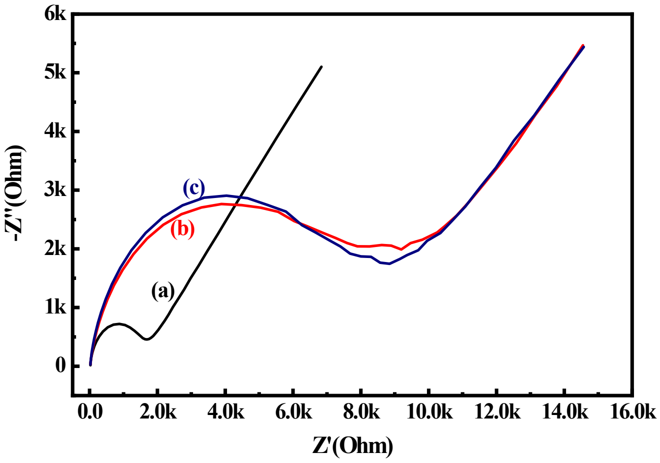

Fig. S5. EIS responses of (a) GCE, (b) PoPD modified GCE and (c) PoPD modified GCE towards 10 μL of 72 mg dL^{-1} aqueous glucose in 25 mL aqueous KCl (1 M) electrolytes containing 10^{-3} M $[Fe(CN)_6]^{3-/4-}$redox couple

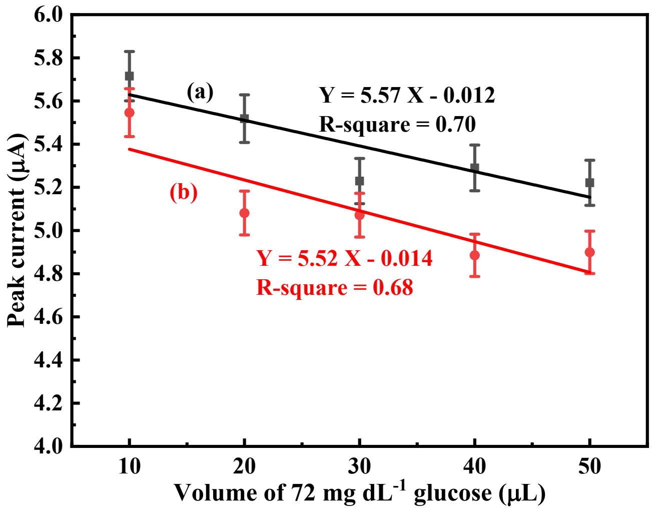

Fig. S6. Linear fitting plot of (a) anodic and (b) cathodic peak current against the volume of glucose

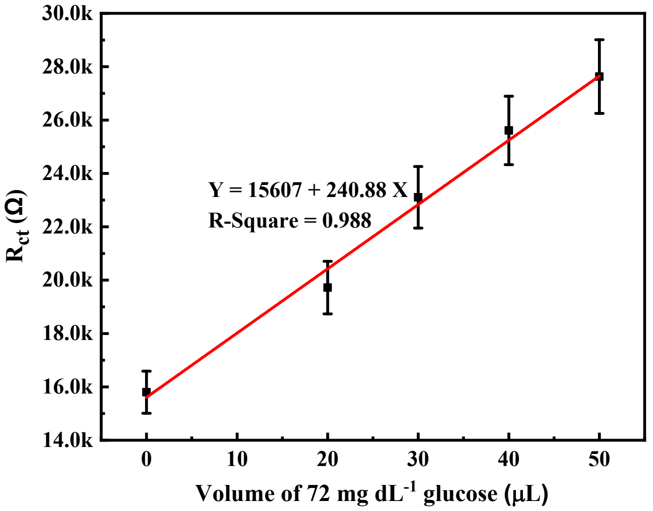

Fig. S7. Linear fitting plot of charge transfer resistance (Rct) value against the volume of glucose

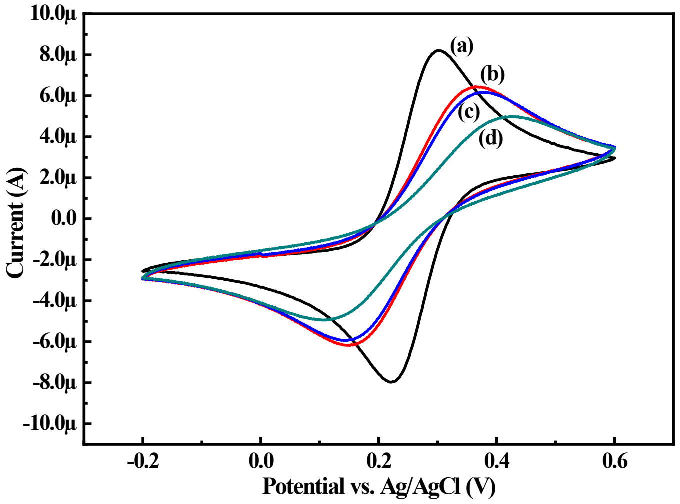

Fig. S8. CV responses of (a) GCE, (b) PoPD/Ag hybrid modified GCE and PoPD/Ag hybrid modified GCE towards (c) 10 µL, (d) 50 µL blood sample in 25 mL aqueous KCl (1 M) electrolytes containing 10^{-3} M $[Fe(CN)_6]^{3-/4-}$redox couple

Table S3. Anodic peak current (I_{pa}), cathodic peak current (I_{pc}), Anodic peak potential (E_{pa}), cathodic peak potential (I_{pc}) and potential difference (ΔE) for the CV responses of blood sample

Sl. no.	Specification	I_{pa} (µA)	I_{pc}(µA)	E_{pa}(V)	E_{pc}(V)	ΔE= E_{pa} -E_{pc} (mV)
01	Clean GCE	8.213	7.971	0.301	0.221	80
02	PoPD/Ag hybrid modified GCE	6.422	6.171	0.365	0.151	214
03	PoPD/Ag hybrid modified GCE towards 10 µL of blood	6.157	5.926	0.377	0.147	230
04	PoPD/Ag hybrid modified GCE towards 50 µL of blood	4.971	4.933	0.424	0.111	313

Table S4. Fitted Rct values and different of Rct in EIS measurements of blood sample

Sl. No.	Specification	Electrode modification	R_{ct} (Ω)	+/-(Ω)
01	Clean GCE	Clean electrode	730	5
02	PoPD/Ag hybrid modified GCE	blank	10452	220
03	PoPD/Ag hybrid modified GCE towards 10 µL of blood	10 µl of 100mg human blood	13379	136
04	PoPD/Ag hybrid modified GCE towards 50 µL of blood	50 µl of 100mg human blood	26685	250
